# Visualization of Ray Propagation through Extended Depth-of-Focus Intraocular Lenses

**DOI:** 10.3390/diagnostics12112667

**Published:** 2022-11-02

**Authors:** Isabella D. Baur, Gerd U. Auffarth, Weijia Yan, Grzegorz Łabuz, Ramin Khoramnia

**Affiliations:** David J. Apple Center for Vision Research, Department of Ophthalmology, University of Heidelberg, 69120 Heidelberg, Germany

**Keywords:** intraocular lens, extended depth of focus, presbyopia correction, ray propagation

## Abstract

Extended depth-of-focus (EDoF) presbyopia-correcting intraocular lens (IOL) models differ in their optical design and performance. In the laboratory, we compared the ray propagation and light intensity profiles of four IOLs: the non-diffractive AcrySof IQ Vivity (Alcon Inc., Fort Worth, TX, USA) and two diffractive models, Symfony ZXR00 (Johnson & Johnson Vision, Jacksonville, FL, USA) and AT Lara 829 MP (Carl Zeiss Meditec, Berlin, Germany). A fourth lens, the monofocal AcrySof IQ SN60WF (Alcon Inc.) acted as the control. We projected a 520 nm laser light through each submerged lens in a bath of fluorescein solution. A camera mounted on a microscope captured the light that emerged from the IOL. We recorded the IOLs’ point spread function (PSF) to determine the presence of unwanted visual effects. The ray propagation visualization and light intensity profile of the monofocal control showed one distinct focus, while the AcrySof IQ Vivity demonstrated an extended focus area. We observed two distinct foci with each diffractive IOL. We found a lower level of light spread beyond the PSF center for the AcrySof IQ Vivity compared to the diffractive IOLs. In conclusion, we could confirm the extended range of focus for all the EDoF IOL models. However, the non-diffractive AcrySof IQ Vivity appears to have a smoother transition from a far to an intermediate range. We discuss whether, in clinical use, the higher level of spurious light we found in the diffractive designs may translate into increased dysphotopsia.

## 1. Introduction

Extended depth-of-focus (EDoF) intraocular lens (IOL) models correct aphakia and presbyopia by enhancing the range of vision from far to intermediate, consequently reducing the need for spectacle correction at these distances.

In contrast to other presbyopia-correcting IOLs, like bifocal or trifocal IOLs, EDoF IOLs create one elongated focus rather than several foci while inducing fewer side effects, like reduced contrast sensitivity and dysphotopsia [1].

Manufacturers use different optical principles to create the EDoF effect, making this a relatively heterogeneous group of IOLs. Several EDoF IOLs apply diffractive optical technologies, e.g., the AT Lara 829 MP (Carl Zeiss Meditec, Berlin, Germany) and the Symfony ZXR00 IOL (Johnson & Johnson Surgical Vision, Inc., Jacksonville, FL, USA) [1], or the xact mono-EDoF (Santen Pharmaceutical Co., Ltd., Osaka, Japan) [2]. A small-aperture design has also been utilized to create an extended range of vision in the IC-8 (AcuFocus Inc., Irvine, CA, USA) [1].

Other EDoF IOLs feature non-diffractive designs like the Mini WELL (SIFI, Catania, Italy), which has an aspheric design and enhances the range of vision using spherical aberrations [1]. Another non-diffractive EDoF IOL is the AcrySof IQ Vivity DFT015 (Alcon Inc., Fort Worth, TX, USA) [3].

The heterogeneity implies that different performances and side-effect profiles should be expected from these different EDoF IOLs. This can make it difficult for clinicians to select an appropriate IOL for their patients. The light-pathways visualization we performed in this study may help surgeons to better understand the optical properties of different EDoF IOLs and to choose an IOL that best meets their patients’ needs.

## 2. Material & Methods

### 2.1. Intraocular Lenses

We analyzed a monofocal AcrySof IQ SN60WF, a non-diffractive EDoF, the AcrySof IQ Vivity DFT015 (both Alcon Inc., Fort Worth, TX, USA) and two diffractive EDoF IOLs, the Symfony ZXR00 (Johnson & Johnson Surgical Vision, Inc., Jacksonville, FL, USA), and the AT Lara 829 MP (Carl Zeiss Meditec, Germany). For each model, we tested five samples with all lenses sharing the same refractive power of +20.0 diopters. The specifications for all the IOLs tested are shown in Table 1.

The AcrySof IQ Vivity DFT015 and AcrySof IQ SN60WF IOL share the same platform: they have the same Acrysof material and the same geometry of their haptic design. While the AcrySof IQ SN60WF served as a monofocal control, the AcrySof IQ Vivity features non-diffractive ‘X-Wave technology’ shaping the wavefront to extend the range of vision to the intermediate distance. There are two surface transition elements in the central 2.2 mm of the IOL, one is a slightly elevated plateau (surface transition element 1) of approximately 1 μm, and the other one is a slight curvature change (surface transition element 2).

Surface transition element 1 delays a portion of the wavefront as it passes through the IOL, relative to the more advanced wavefront passing through the IOL outside of the central surface transition elements. The simultaneous actions of the two surface transition elements deliver the extended focal range of the lens [4].

The Symfony ZXR00 and the AT Lara 829 MP are low-add bifocal lenses [5,6] that rely on diffractive technology to create an extended depth of focus effect [5,6,7,8,9]. Both IOLs apply the first- and second-order diffraction for a far and an intermediate focus, which enables a simulation correction of longitudinal chromatic aberration at both foci [7,8,9].

The Symfony combines diffractive and refractive technologies: the posterior surface features an embedded echelette design that elongates the range of vision and lowers chromatic aberrations [5,8]. The Symfony’s secondary focus occurs at about 1.75D [5,8] and its aspheric anterior surface compensates for corneal spherical aberration [10].

The AT Lara also features a diffractive design creating two foci, one for intermediate distance with a net addition power of +1.9 diopters and one for far distance [6]. The aspheric optical design is aberration neutral and optimized for the correction of chromatic aberrations [11,12].

### 2.2. Ray Propagation Setup

We used an experimental setup that was similar to the one described in one of our previous studies [13], with a light source, condenser, beam expander, model cornea, and a water bath.

**Figure 1:**
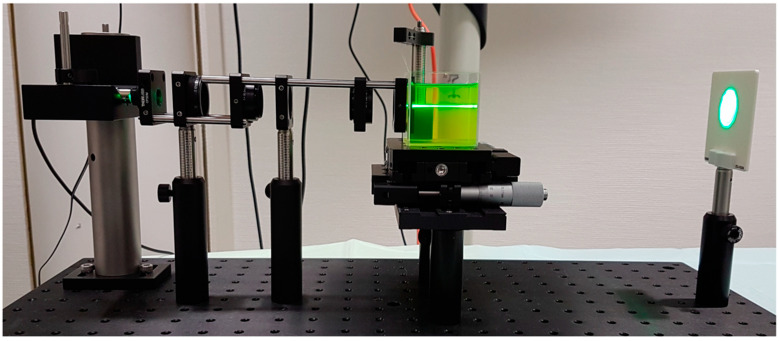
Setup for ray propagation imaging. From left to right, the following setup components are depicted: a monochromatic green laser light source (520 nm) projects through a diaphragm and a beam expander (f1 = 30 mm and f2 = 60 mm). A model cornea is a plano-convex lens (f = 30 mm) placed between the illumination system and the water bath containing a fluorescein solution. An IOL holder is submerged in the fluorescein solution, and a screen is mounted behind the water bath.

The IOL under test was placed in a lens holder with a 3.0 mm aperture and immersed in the water bath, which was stained with a fluorescein solution in a concentration of 100 mg/mL. The monochromatic green laser light source, with a wavelength of 520 nm, was clamped onto a V-mount and projected through the model cornea and the IOL. The model cornea we used was a plano-convex lens with a focal length of 30 mm. This setup allowed us to visualize the light distribution, which was captured using a camera that was mounted onto a surgical microscope (Leica, Wetzlar, Germany) with a 40× magnification. The images were then analyzed using ImageJ software to obtain the pixel intensity values along the IOL’s optical axis. The direction of the light propagation was set as the reference point to determine the position of each focus. The setup is depicted in Figure 1.

### 2.3. Unwanted Visual Effects Testing

Halos are positive dysphotopsia that oftentimes result from a superimposition of several images on the retina. Therefore, they occur more frequently with multifocal IOLs that have several foci than with monofocal IOLs that have one focus [14,15].

We used the OptiSpheric IOL PRO2 (Trioptics GmbH, Wedel, Germany) to assess the unwanted visual effects of the test lenses and the monofocal control. This device complies with the International Standard Organization recommendations [16] and includes a polychromatic light source, spectral filters, a test object, a collimator, a corneal model with +0.28 µm of spherical aberration, an IOL holder, a microscope objective, and a charged-coupled device (CCD) camera. We chose this model cornea as it simulates the mean spherical aberration found in the population studies [17]. The IOL was submerged in a balanced salt solution in the IOL holder during the testing.

A 0.1 mm pinhole was illuminated by a collimator and imaged by the IOL onto the CCD camera [18]. We used a 4.5 mm aperture in this setup to compare the light distribution beyond the point spread function (PSF) center of the different IOL models.

## 3. Results

**Figure 2:**
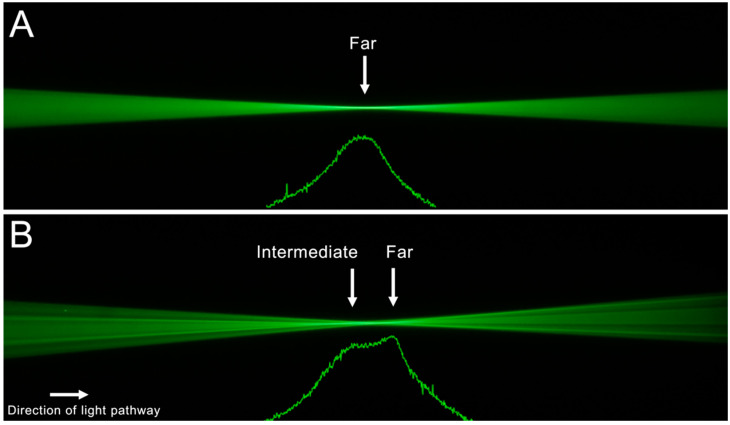
(**A**): Light-pathways visualization and light intensity profile of the monofocal control IOL SN60WF with one distinct focus. (**B**): Ray propagation and light intensity profile of the Acrysof IQ Vivity IOL with an elongated focus.

**Figure 3:**
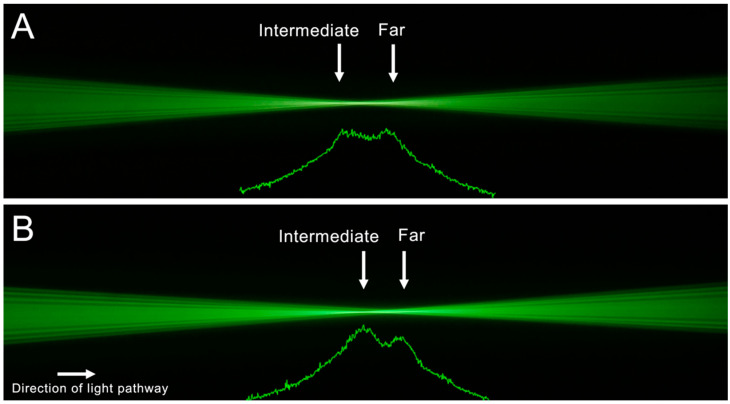
Light-pathways visualization and light intensity profile of the AT Lara (**A**) and Symfony IOL (**B**). Both IOLs showed distinct foci for far and intermediate distances. In the Symfony, a minimally higher light intensity value was found for the intermediate compared to the far distance.

Figure 2 and Figure 3 show the ray propagation images from left to right. Below each light-pathways visualization, the corresponding light intensity profile is depicted. This profile shows a relative pixel value. For the monofocal IOL (Figure 2A), we observed one focus corresponding to the nominal lens power. For the Acrysof IQ Vivity IOL (Figure 2B), the visualization of the ray bundles and the intensity profile both revealed an elongated focus area. The results for the other two EDoF IOLs are shown in Figure 3A,B. Both IOLs showed distinct foci for far and intermediate distances. While for the AT Lara (Figure 3A), both foci showed similar high peaks of the intensity profile; for the Symfony IOL (Figure 3B), we observed a higher peak for the intermediate distance.

### Unwanted Visual Effects

**Figure 4:**
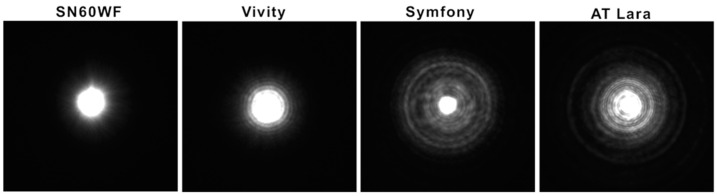
The PSF images present a visualization of the light distribution with a 0.1 mm pinhole for all IOLs tested. Both diffractive IOL models created an extended halo pattern. The Vivity showed minimally increased light spread compared to the monofocal SN60WF.

The results of the unwanted visual effects testing are shown in Figure 4. Compared to the monofocal lens, the light spread is minimally extended for the Acrysof IQ Vivity. The diffractive lenses both produced several halo-like rings with an excessive light intensity around the center of the PSF.

## 4. Discussion

EDoF IOLs provide a functional vision from far to intermediate distance [2,19,20,21,22,23,24,25,26]. There are reports that certain models are associated with a lower level of dysphotopsia than other presbyopia-correcting IOLs [2,3,24]. However, as the EDoF effect is not precisely defined, it may be difficult to predict the performance of a specific device labeled as an ‘EDoF’ IOL. In an attempt to standardize the classification, the American Academy of Ophthalmology released a consensus statement defining the criteria for the minimum performance levels an IOL should achieve to be labeled as ‘EDoF’ [27]. The visualization of an IOLs’ light pathways may help to better compare the function of different EDoF IOLs.

The ray propagation method we used relies on the fluorescent reaction of the fluorescein solution but also on a certain amount of light scattering as we used a laser with a 520 nm wavelength, which differs from the optimal excitation wavelength of fluorescein of 490 nm [28], and therefore requires higher laser intensities to be applied. The resulting image is composed of fluorescence and scattered excitation light. Instead of using fluorescein, one can use other media to create light scattering to visualize the light pathways: Eppig et al. [29] used the alcoholic beverage Ouzo. The image quality suffered from oil droplets in the Ouzo solution, which hampered the quantitative evaluation of the images. They found fewer image artifacts with a fluorescein solution when creating images making use of only the fluorescent properties. They used a different wavelength of 405 nm for the excitation of the fluorescein. This approach requires less laser intensity and leads to less light scattering that could compromise the image quality and it also allows one to create an image solely from the emitted light using filters to eliminate the exciting light [29]. As we obtained a satisfactory image quality with our setup, we conclude that the fluorescein solution is a more suitable medium than an Ouzo solution for ray propagation imaging and that the laser source we used seems appropriate for obtaining images at a sufficient quality to perform a quantitative analysis. Following the internal evaluation of various media, the one with fluorescein provided the highest quality of ray-propagation visualization. So, the potential advantage of the Ouzo solution is that it may be used for measurements with different wavelengths, as this method does not depend on the excitation wavelength in contrast to the fluorescein method [29]. The wavelength we used is also closer to the retina’s maximum sensitivity of 555 nm, which makes it more interesting than the wavelength of 405 nm, which is close to ultraviolet light and to which the retina has a ten-fold less sensitivity [13,29]. Additionally, the optical performance of diffractive IOLs changes when a different design wavelength (e.g., 550 nm) is used in the assessment [7,9,30]. Diffractive EDoF IOLs featuring the second-order design have increased the spectral dependency of their diffraction efficiency. As a result, the IOLs act similarly to a monofocal lens when illuminated by blue (e.g., 480 nm) or red (e.g., 644 nm) light, and this characteristic may affect the patients’ visual function, as shown clinically [31].

Apart from visualizing the focal points of an IOL, ray propagation imaging can also be used to determine the focal length of an IOL [13,32]. It has also been used to successfully visualize straylight caused by IOL opacifications In a study by Son et al., scattered light caused by calcification or glistenings in the IOL material could be visualized after an appropriate post-processing of the images [33].

The light-pathways visualization and light intensity profile showed one distinct focus for the AcrySof IQ SN60WF IOL, confirming its monofocal properties. The ray propagation imaging of the different EDoF IOL models demonstrated an extended range of focus when compared to the monofocal control. However, only the non-diffractive model presented a more uniform light-intensity transition from far to intermediate. By contrast, two distinct foci were observed for the diffractive IOLs. While the non-diffractive Vivity and the diffractive AT Lara showed a far-dominance in their light intensity curves, the Symfony showed a higher light intensity for the intermediate focus, which is in agreement with earlier reports [5,7]. It has to be considered, though, that we only performed the ray propagation imaging using a 3.0 mm aperture and it has been previously shown that the Symfony shows a more far-dominant behavior when testing is performed using a 4.5 mm aperture [13].

In an earlier work, our group found a good agreement between ray propagation imaging and the through-focus modulation transfer function (MTF), which shows the important connection between the distribution of light energy and image quality [13]. The results for through-focus MTF measurements have been published for all the IOL models we assessed in this present study and are in a good agreement with the results we found for the ray propagation imaging and the light intensity profiles: the monofocal AcrySof IQ SN60WF showed one distinct peak, as expected, for a monofocal IOL [13]. For the Acrysof IQ Vivity IOL, only one peak was seen in the through-focus response at the 3 mm aperture, with an extended depth of focus of 2.20 diopters [34]. For the Symfony and the AT Lara, however, it was reported that the through-focus MTF curves showed two peaks for the 3 mm pupil size [5,6].

All three of the EDoF lenses we tested were also assessed in clinical studies. For the Symfony, a meta-analysis by Liu et al. [35] found a superior intermediate visual acuity compared to a monofocal control and a better contrast sensitivity compared to trifocal IOLs. There were no differences in the spectacle independence or the occurrence of dysphotopsia compared to trifocal IOLs [35]. For the AT Lara, the visual acuity results as well as the results of the dysphotopsia assessment published are comparable to those of the Symfony [36,37]. The AT Lara was superior to the Symfony at monocular defocus curve testing [37]. For the Acrysof IQ Vivity, good visual acuity results for far and intermediate distance and functional visual acuity for near distance were reported [3,38]. Additionally, a low level of photic phenomena was reported for this IOL model [3,38].

These clinical data are supported by our results from the analysis of unwanted visual effects: with the Acrysof IQ Vivity showing minimal spurious light compared to the monofocal IOL, while the diffractive IOL models show halo-like photic phenomena.

It has been previously shown that the PSF images correspond to the clinical findings. For the ICB00 and ZCB00, comparable levels of photic phenomena were observed clinically. This was confirmed by the PSF, which showed an only 8% larger halo compared to a monofocal IOL for the ICB00 IOL [18]. However, it has also been shown that the perception of dysphotopsia does not depend on the optics of the IOL implanted alone. An existing refractive error can apparently also lead to an increased perception of photic phenomena. It has been previously reported that patients who underwent a monovision treatment reported more photic phenomena than patients with an emmetropic target refraction in both eyes [39]. However, compared to the monovision treatment, the implantation of a bifocal diffractive IOL resulted in an increased level of dysphotopsia [40]. Therefore, the perception of bothersome photic phenomena can only be predicted to a limited extent and must always be verified in clinical studies.

Light scattering at diffractive steps may fall into a larger angular range, which cannot be accurately quantified using the current setup. A further study is required to assess the contribution of a diffractive profile to glare phenomena.

It has been reported that IOLs without spherical aberration correction show blurrier focal points in their light pathways [32,41]. All lenses assessed in this study correct corneal spherical aberrations, but each to a different extent. However, as we used a 3 mm aperture through which to propagate the rays, we consider that the spherical aberration effects are negligible.

A limitation of our study is that we used a monochromatic green light for the ray propagation imaging. In vivo, light sources usually are composed of multiple wavelengths. To provide a more realistic image of the in vivo performance, we should consider for future studies to develop a method to evaluate the IOL performance under polychromatic light.

Another limitation is that we did not include the successor model of the AcrySof IQ Vivity, the Clareon Vivity (Alcon, Fort Worth, TX, USA), which is made of a glistening free material, in our analysis. However, the availability of this version of the IOL is currently limited to certain countries.

## 5. Conclusions

Ray propagation imaging is a useful adjunct to other objective assessments on the optical bench as well as clinical studies to provide the clinician with a better understanding of how different intraocular lenses work. In our study, all the EDoF lenses showed an extended visual range. The non-diffractive EdoF lens demonstrated one continuous elongated focus, while the diffractive models produced two distinct foci. The unwanted visual effects testing revealed a lower level of spurious light for the non-diffractive EdoF IOL, suggesting that this IOL may induce less halos than the diffractive IOL models.

## Figures and Tables

**Table 1:** Key characteristics of the tested intraocular lenses.

	AcrySof IQ SN60WF	AcrySof IQ Vivity DFT015	Symfony ZXR00	AT Lara 829 MP
Optic design	One-piece	One-piece/wavefront shaping-optic	One-piece/combined diffractive-refractive extended-depth-of-focus	One-piece/combined diffractive-refractive extended-depth-of-focus
Lens diameter	13.0 mm	13.0 mm	13.0 mm	11.0 mm
Optic diameter	6.0 mm	6.0 mm	6.0 mm	6.0 mm
Dioptric power	20.0 D	20.0 D	20.0 D	20.0 D
Lens material	Hydrophobic Acrylate	Hydrophobic Acrylate	Hydrophobic Acrylate	Hydrophilic Acrylate (25% water content), hydrophobic surface
Refractive index	1.55	1.55	1.47	1.46
Spherical Aberration	−0.20 μm	−0.20 μm	−0.27 μm	Aberration neutral

## Data Availability

Data are contained within the article.
